# Co-regulation of a large and rapidly evolving repertoire of odorant receptor genes

**DOI:** 10.1186/1471-2202-8-S3-S2

**Published:** 2007-09-18

**Authors:** Marijo B Kambere, Robert P Lane

**Affiliations:** 1 Department of Molecular Biology and Biochemistry, Wesleyan University, Middletown, CT, USA

## Abstract

The olfactory system meets niche- and species-specific demands by an accelerated evolution of its odorant receptor repertoires. In this review, we describe evolutionary processes that have shaped olfactory and vomeronasal receptor gene families in vertebrate genomes. We emphasize three important periods in the evolution of the olfactory system evident by comparative genomics: the adaptation to land in amphibian ancestors, the decline of olfaction in primates, and the delineation of putative pheromone receptors concurrent with rodent speciation. The rapid evolution of odorant receptor genes, the sheer size of the repertoire, as well as their wide distribution in the genome, presents a developmental challenge: how are these ever-changing odorant receptor repertoires coordinated within the olfactory system? A central organizing principle in olfaction is the specialization of sensory neurons resulting from each sensory neuron expressing only ~one odorant receptor allele. In this review, we also discuss this mutually exclusive expression of odorant receptor genes. We have considered several models to account for co-regulation of odorant receptor repertoires, as well as discussed a new hypothesis that invokes important epigenetic properties of the system.

## Introduction

**Figure 1:**
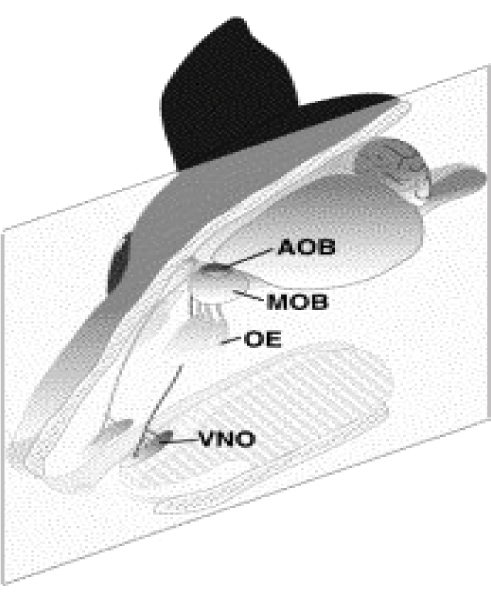
**Schematic of the olfactory and vomeronasal organs in rodents.** The olfactory epithelium (OE) contains the olfactory sensory neurons, which project their axons to the main olfactory bulb (MOB). The vomeronasal organ (VNO) contains the vomeronasal sensory neurons, which project their axons to the accessory olfactory bulb (AOB). Figure reprinted from [127], Copyright (2005), with permission from Elsevier.

Animals depend on chemosensory systems to investigate their environments and to communicate social and reproductive status. In the mammalian olfactory system, two anatomically distinct organs allow these functions: the nose and the vomeronasal organ (VNO) (Fig. 1). The main olfactory system (the nose) consists of an olfactory sensory neuronal epithelium that detects odorant molecules and transduces odorant information to the olfactory bulb of the brain, where it is processed into the perception of smell. The vomeronasal organ (VNO), located just above the roof of the mouth in most mammals, is thought to be largely responsible for the detection of pheromones that provide subconscious information about the social and sexual status of individuals within the same species.

The ability to detect odorants in these two chemosensory organs in mammals is mediated by four families of G-protein-coupled receptor (GPCR) genes. The olfactory sensory neurons (OSNs) of the main nose express two of these families, the olfactory receptor (OR) gene family [1] and the trace amine-associated receptor (TAAR) gene family [2]. The vomeronasal neurons of the VNO express the other two families, named V1R [3] and V2R [4-6]. While the ORs and TAARs couple to the same G-protein [2,7,8], the V1R, V2R, and OR/TAAR genes utilize distinct signal transduction pathways [9] and are expressed in anatomically and functionally distinct types of sensory neurons.

How does the brain make sense of a complex odorant world? The repertoires of odorant receptors must be sufficiently large and diverse to detect and distinguish among a vast array of odorant structures, the chemosensory organs and processing centers of the brain must be designed to integrate sensory neuronal activity into coherent "smells", and both the receptor repertoires and the brain must keep pace with changing olfactory requirements associated with niche development and speciation. In this review, we describe the evolution and genome organization of odorant receptor repertoires in various species, as revealed by comparative genomics, to discuss the functional range and adaptive ability of the olfactory system. We also describe the regulation of the expression of these odorant receptor repertoires during development, since, as we will discuss, OR co-regulation is the basis for an organizing principle that permits the brain to interpret smells. Finally, we consider a possible relationship between these two topics – that OR co-regulation might depend on genome organization, as it does for some other large, clustered receptor families in the mammalian genome.

## Large and divergent odorant receptor repertoires reflect niche and species specificity

### Olfactory receptor repertoires of the main olfactory system

**Figure 2:**
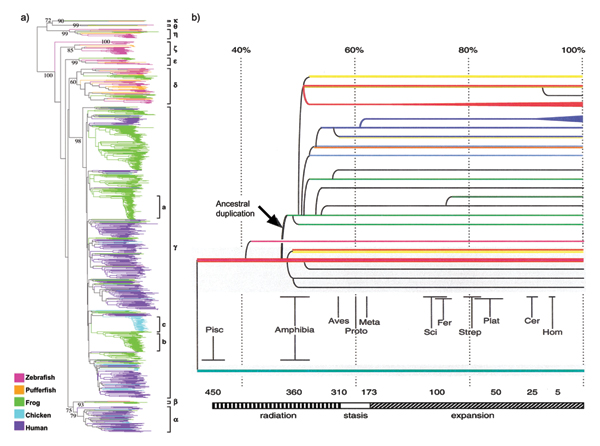
**Vertebrate olfactory receptor gene phylogeny. a) **Phylogenetic tree of all intact olfactory receptor genes identified from zebrafish (102 genes), pufferfish (44 genes), *X. tropicalis *(410 genes), chicken (82 genes) and human (388 genes). Branches are color-coded by species (see key). The nine major clades are labeled (α, β,γ, δ, ε, ζ, η, θ, κ); human fish-like Class I genes are within the α clade and human Class II genes are within the γ clade. Vertebrate Class II repertoire expansions include both old (e.g., human) and recent (e.g., frog and chicken; clades indicated by a-c) gene duplications. Bootstrap values are indicated at branch nodes. Figure from [15]. **b) **Schematic tree illustrating coalescence of human OR families (color coded branches) to the period of evolution when humans and amphibia diverged, ~370 MYA. Percent divergence is translated to divergence time by applying molecular clock estimates. Taxonomy: Pisc = fish; Amphibia = frogs, salamander; Aves = chicken; Proto = platypus; Meta = koala; Sci = marmot, mouse, rat; Fer: pig, dolphin, dog; Strep = lemurs, squirrel monkeys, Plat = marmoset; Cer = baboon, macaques; Hom = chimpanzee, gorilla. Figure reprinted from [19], Copyright (2001), with permission from CSH Press.

The olfactory receptors (ORs) of the main nose comprise the largest gene family in the mammalian genome. Terrestrial mammals possess a repertoire that is approximately 1,000 OR genes. According to current counts, the mouse genome encodes at least ~1400 OR genes [10], the canine genome encodes at least ~1000 ORs [11], the chimpanzee genome encodes at least ~1000 ORs [12], and the human genome encodes at least ~800 ORs [13]. OR repertoires in fish are approximately 10-times smaller: zebrafish has 143 genes, fugu 44 genes, and tetraodon 42 genes [14]. Nevertheless, the relatively small OR repertoires in fish species are more diverse than the large mammalian repertoires [14,15] (Fig. 2a). In mammals, all ORs fall into two major groups, based on sequence homology. These are termed "Class I" (~10% of the repertoire) and "Class II" (~90% of the repertoire). Fish ORs fall into >5 groups based on this same criterion. Therefore, the fish OR repertoires, while much smaller, probably respond to much more divergent kinds of odorants; the much larger but less diverse mammalian repertoires on the other hand, will likely do better at discriminating between structurally similar odorants.

Interestingly, the smaller family of Class I ORs in mammals are described as "fish-like", because they share greatest sequence homology with fish OR families [15-19] (Fig. 2a). This observation raises the possibility that less "fish-like" Class II OR repertoires are more recent adaptations to life on land. Consistent with this hypothesis, the Class II OR repertoires in mammals coalesce to about the time of the common ancestor with amphibians (Fig. 2b). Moreover, the amphibian *X. laevis *exclusively expresses Class I ORs in the water-filled diverticulum of their nasal cavities, and expresses Class II ORs in the air-filled diverticulum of their nasal cavities [16]. These observations suggest that when our ancestors began to occupy niches on land, much of the current diversity seen in the mammalian Class II repertoires arose, and these more recently evolved ORs were adapted for the detection of airborne (or volatile) odorants [17,19].

The invertebrate odorant receptor repertoires identified in the fly [20-22] and worm [23,24] genomes share no sequence homology to each other or to those in vertebrates. The chemosensory receptor repertoire in *Caenorhabditis elegans*, like mammals, is large (>800 ORs) and diverse (five distinct families). In contrast, the *Drosophila melanogaster *genome encodes only a single family of ~60 OR genes. Therefore, the absolute size of OR repertoires does not seem to correlate with behavioral sophistication (worms and humans have repertoires that are ~10-times the size of flies and fish). Also, the lack of homology between odorant receptor repertoires of invertebrates and vertebrates suggests that the evolutionary requirement for distinct olfactory abilities in these species is met by recruitment of novel gene families rather than exploiting preexisting families in ancestral genomes.

### V1R and V2R repertoires of the vomeronasal organ

The vomeronasal receptor (VR) gene familes, V1R and V2R, expressed in the second mammalian chemosensory organ, the VNO, also exemplify independent gene family recruitment during evolution. These two families share no sequence similarity to each other, nor to any of the numerous, diverse OR families described above. Complete V1R repertoires have been described in a number of mammalian species, including mouse, rat, opossum, dog, cow, chimpanzee, and human [10,25-29], as well as five fish species [30]. The mouse and human V1R repertoires were the first to be characterized, and the >150 putative functional V1Rs in mouse seemed likely to be representative of mammals, whereas the merely ~5 intact V1Rs in humans seemed exceptional. This perspective was reasonable, since mouse, like most mammals, makes extensive use of their olfactory systems for social and reproductive communication, whereas humans do not seem to be as reliant on these forms of communication. Consistent with this view, the human VNO is probably non-functional [31]. Moreover, the human TRP2 ion channel, required for mouse VNO function, is a pseudogene [32-34], and at least one of the intact human V1Rs is expressed in the main olfactory epithelium [35]. Surprisingly, however, subsequent characterization of other mammalian V1R repertoires suggests that the large functional repertoire in rodents may be more exceptional. Chimpanzee has no intact V1R genes [29], and dog, cow, and opossum have only 8 [29], 32 [28], and 49 [28] intact V1R genes, respectively. Rat, on the other hand, has at least 107 intact V1Rs [29]. These observations suggest that rodents may have evolved specialized and more complex functions for the VNO than these non-rodent mammalian species. Consistent with this perspective, the morphological complexity of the rodent VNO is proportionately greater than other mammalian species [28,36].

The large rodent V1R repertoires consist of at least 12–13 distinct subfamilies [25]. The duplication events that gave rise to these divergent subfamilies probably took place prior to mammalian divergence, because each of these 12–13 subfamilies is represented in the large V1R pseudogene content of other mammals [29]. Thus, it is proposed that most of the ancestral V1R functions were lost in mammals, except for in rodents where, in contrast, many of these ancestral functions have expanded by extensive gene duplications. Of note is the identification of a single V1R-like sequence in fish [30], which is expressed along with ORs in their single chemosensory organ (fish do not possess a VNO). Although V1Rs have not yet been characterized in amphibians, it is tempting to speculate that V1R diversification, like Class II OR diversification, occurred in vertebrate ancestors as they adapted to land, and evolved a separate VNO that could support more sophisticated pheromonal behaviors, like territorial marking and reproductive display (e.g., [37-39]).

Some of the mouse and rat V1R subfamilies delineate along species lines. Striking examples include two subfamilies in which all of the mouse and all of the rat genes partition into separate clades of a phylogenetic tree [26], suggesting post-speciation expansion and specialization, and two subfamilies in which the rat genes have been deleted [29], suggesting species-specific capabilities exclusive for mouse. Similar lineage-specific diversification of mouse and rat V2R subfamilies is observed [40]. Interestingly, duplication events that produced species-specific delineations in some of the V1R subfamilies have been dated to a short period of evolution approximately when mice and rat speciated [26], suggesting that this diversification might have been favored to reinforce species divergence. These examples suggest strong delineation of VNO function between these two rodent species. Species-specificity in V1R and V2R repertoires could be part of an explanation to account for how pheromone communication occurs within but not between species.

Mouse and rat V2R receptor repertoires are approximately the same size as their V1R repertoires and composed of ~100 intact genes [40]. A recent analysis of several mammalian genome assemblies suggests that V2Rs, like V1Rs, are more prominent in rodent than non-rodent mammals, with opossum being an exception. The comparatively large opossum repertoire of ~49 intact V1R genes [28] and ~90 intact V2R genes [41] appears to be consistent with the presence of a well-developed vomeronasal organ in this marsupial mammal. In fish, V2R repertoires tend to be much larger than V1R repertoires, and comparable to their OR repertoire sizes [42]. As observed for rodent gene families, fish V2R evolution includes lineage-specific expansions, presumably to support adaptations to conspecific small-peptide cues [43].

In summary, the olfactory systems of insects, worms, fish, and mammals utilize at least nine, evolutionarily distinct odorant receptor gene families adapted to their very different niches. In vertebrates, the defining evolutionary moment occurred as amphibian-like ancestors adapted to land, when the Class II ORs expanded and diversified. The repertoires of trace amine-associated receptors and putative pheromone receptors, V1R and V2R, are at least ten-times smaller than the OR repertoire sizes in all mammalian species so far characterized. V1R and V2R repertoire size and diversity, like VNO morphological complexity, is highly variable among mammals, and these differences might contribute to species-specificity in VNO function. We next consider evolutionary mechanisms that have contributed to species specific adaptations in odorant receptor repertoires.

## Adaptive evolution of odorant receptor repertoires

Even before genomes were sequenced and complete odorant receptor repertoires had been characterized, the dynamic evolutionary history of odorant receptor genes was evident. Fluorescence *in situ *hybridization using specific OR probes on metaphase human chromosomes revealed patterns of very recent duplications and chromosomal translocations [44,45]. Strikingly, some of these OR gene duplications appear to have occurred so recently that they produce copy number polymorphisms in the human population [46]. These observations suggest that OR repertoires are not fixed in populations, and that the olfactory system is constantly generating new genes that are likely to underlie adaptive evolutionary innovations.

### Gene duplication and gene conversion

The OR repertoire in terrestrial vertebrates has expanded approximately 10-fold since the common ancestor with aquatic fish, approximately 370 million years ago. This wave of expansion is believed to reflect adaptations of the olfactory system to meet new requirements and opportunities in non-aquatic environments [19]. The earliest duplication events gave rise to the major families of Class II ORs found in all mammals. Following this initial phase of expansion, individual OR subfamilies have expanded locally to generate large gene clusters [19,47-50]. Segmental duplications between chromosomes have also contributed to wide distribution of OR clusters, which are found on nearly every chromosome in the mouse and human genomes [44,45,49]. These and other comparative genomics studies have revealed some general principles about OR evolutionary mechanisms, that also seem to apply to TAAR, V1R, and V2R evolution [10,26-29,40,51-55]. First, tandem duplications, presumably mediated by unequal crossover during meiosis, have resulted in a predominance of relatively homogenous clusters; that is, tandem arrays of closely-related genes (e.g., [10,19,47]). Second, gene conversion and recombination events generate mosaic receptor sequences, and potentially, might contribute to the re-birth of OR pseudogenes during evolution [56]. Third and alternatively, gene conversion of entire coding regions can result in homogenization whereby the overall diversity of the OR and VR repertoires is reduced via replacement of one paralogous sequence with another [56]. Fourth, duplication events have probably been facilitated at OR and VR loci by retrotransposon activity. The dense populations of retrotransposons within OR and VR gene clusters indicates a history of frequent DNA breaks during retrotransposition, as well as increased opportunity for repeat-mediated misalignments to cause unequal crossovers during meiosis [19,26,50,52,57]. Overall, evolution of OR and VR genes has been a dynamic process of expansion, diversification, and modulation, resulting in the generation of large tandem clusters that are widely distributed in the genome.

### Adaptive selective pressures acting on duplicated ORs

Since mutations occur randomly, and therefore equally in silent and non-silent positions of a gene, an observed bias for silent mutations (that do not alter amino acid code) or non-silent mutations (that alter amino acid code) indicates whether selection has favored stasis or adaptation. The *Ka:Ks ratio*, a ratio of the rate of non-synonymous (amino-acid changing) substitution to the rate of synonymous (or silent) substitution in coding sequences, is used in this way to assess selective forces acting on gene pairs since their common ancestry. Sequences under purifying (or negative) selection will exhibit Ka:Ks ratios significantly less than 1 (i.e., a much higher rate of synonymous substitution); most gene sequences are under purifying selection, because most often, genes are well adapted and fixed, thus changing the amino acid code is selected against. Sequences under adaptive (or positive) selection will exhibit Ka:Ks ratios significantly greater than 1 (i.e., a much higher rate of non-synonymous substitution), indicating that selection is acting against genes that do not evolve new functions. Sequences not subject to selective pressure (neutral sequences) will exhibit Ka:Ks ratios of approximately 1, indicating that neither silent nor non-silent mutations have been favored.

**Figure 3:**
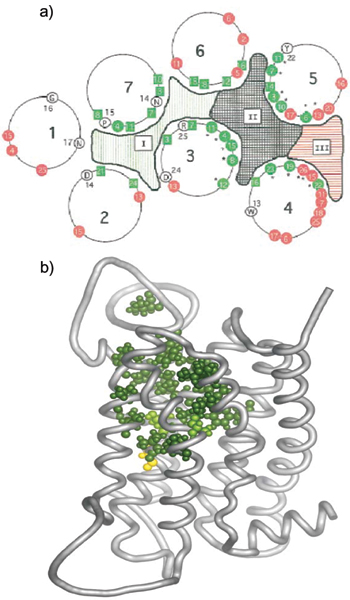
**Predicted structures of olfactory receptor proteins modeled against the rhodopsin GPCR, depicting putative ligand-binding pockets between helical transmembrane (TM) domains**. **a) **Schematic depicting an overhead view of the seven transmembrane-spanning barrels of an OR protein. The residues in each helix are numbered separately, according to the predicted TM boundaries. Residues conserved among all GPCRs are shown in open circles. Colored squares and circles represent positions of conserved and variable residues, respectively. Residues that align with ligand contact residues in other GPCRs are colored green and residues that do not align with these residues are colored red. Hypervariable residues (putative odorant-binding residues), which are thought to be under positive selection, are indicated by asterisks. Area II denotes a hypervariable pocket that corresponds to the ligand binding pocket in other GPCRs. Figure modified from [61]. **b) **Side view of a predicted OR protein structure as seen from within the membrane. Putative OR ligand-binding residues are shown in green and yellow (green : based on homology with non ORGPCR; yellow based on OR evolutionary analysis). Figure from [128].

Analysis of OR, V1R, and V2R sequences reveals that different portions of the gene are exhibiting different selective pressures. For example, analysis of 136 intact zebrafish OR genes suggest that four specific sites within the third and fourth transmembrane domains (TM3, TM4) and the third extracellular loop of these GPCRs is under adapative selection, whereas the remainder of the coding sequence is under purifying selection [14]. Similar studies conducted on sets of mouse, rat and fish OR genes point to specific sequences encoding residues in transmembrane domains that exhibit high Ka:Ks ratios [53,58]. While no crystal structure of an OR or VR protein has been solved, homology modeling to the rhodopsin [59] or metabotropic glutamate receptor [60] GPCR structures and their ligands, as well as energy minimization modeling of receptor-ligand interactions, suggest that the hydrophobic surfaces of TM 3–7 in mammalian ORs is likely where ligand binding occurs (e.g., [61-64]). Specific residues predicted to lie within these putative hydrophobic ligand-binding spaces generally appear to be hypervariable in evolution (Fig. 3). Similar observations have been made for V1R and V2R subsequences [40,52,53,55,65]; in fact, Ka:Ks ratios are generally higher in V1Rs than ORs [10], possibly reflecting increased pressures to adapt independent pheromone responsiveness during speciation. Like ORs, hypervariable residues in V1Rs and V2Rs correspond to areas of the protein structure predicted to be within putative ligand-binding domains [40,53]. These data suggest selection for duplicated ORs, V1Rs, and V2Rs to adapt new ligand-binding properties, possibly favored in order to meet the demands and opportunities of new niches and speciation.

### OR polymorphisms within populations

The selective forces that have favored adaptive changes in OR and VR proteins between species also seem to operate within species. Like antigen receptor genes of the immune system, the odorant receptor genes of the olfactory system exhibit high allelic variation in the population [46]. Allelic variation probably accounts for observed differences in olfactory perception among individuals in the human population [66,67] and between mouse strains [68,69], although genetic variation in any of the peripheral and central components of the olfactory system must also be considered. It has been estimated that the incidence of single nucleotide polymorphisms (SNPs) in mouse OR gene coding regions is about twice that of other mouse GPCRs [10]. Studies in two additional species, humans and dogs, illustrate the biological significance of OR variation. Comparison of 32 OR loci in human subpopulations illustrates that, for example, Caucasians tend to have a higher frequency of non-functional alleles as compared to Pygmy populations [70]. Another study of 51 human highly polymorphic OR loci came to a similar conclusion that functional and non-functional alleles correlate with ethnicity [71]. It is tempting to speculate that variation of ORs in human subpopulations was due to adaptation to different environments and diets during human migration. Dog ORs, like human and mouse, are highly polymorphic. In a study of 16 OR genes in 20 different breeds of dogs, >50% of the SNPs (55 of 98 polymorphisms) generated an amino acid change, and several alleles appear to be breed specific [72]. Given the significant differences in abilities that have been selected in various dog breeds, it will be interesting to further investigate the extent to which allelic variation correlates with breeds whose domestic functions rely more heavily on olfaction.

Genetic variation in OR repertoires might not only be due to polymorphic nucleotide substitutions, but in addition, to copy number polymorphisms. Copy number polymorphisms arise by recent gene duplications that render some individuals in the population with "extra OR copies". For example, the cluster of ORs found in the subtelomeric regions of chromosome 19 has variable duplication histories in the human population [46]. Analysis of 45 individuals from eight ethnic groups reveals that 7–11 copies of this cluster are identified at subtelomeres of other chromosomes. The biological significance of these copy number polymorphisms is unknown.

### Pseudogeneization and decline of olfactory functions

To this point we have discussed "gene birth": duplication and adaptation as evolutionary mechanisms that have permitted animals to expand into new environmental territories and diverge during speciation. The other side of this evolutionary process is "gene death". Non-functional OR, V1R, and V2R pseudogenes are abundant in mammalian genomes, and especially so in primate genomes. There is a lower percentage of Class I pseudogenes than Class II pseudogenes in human (52% versus 77%), dog (17% versus 23%), and rat (13% versus 20%) [19,73]. Class I genes have also not undergone extensive duplications like the Class II repertoires.  These trends might indicate that some ancestral fish-like odorant-binding functions have been fixed in mammalian genomes.

The human genome contains ~800 OR genes, yet >50% of these are pseudogenes [19,74]. In contrast, of the ~1400 mouse OR genes, only ~20% are pseudogenes [47,69]. Even compared to other primate species, human OR coding regions have accumulated pseudogenes ~4-times faster than chimpanzee, gorilla, orangutan, and macaques [75]. Nevertheless, the subfamily representation in the intact repertoire of human ORs is the same as in mouse [47,76], suggesting that humans are able to smell as broad a range of odorants as other mammalian species, but have probably lost some discriminatory ability. It has been proposed that the decline in human olfactory ability might have been concurrent with bipedalism and the advent of a dominant visual system (e.g., [76-78]).

The V1R "death" process is even more extreme than the decline of ORs in primate lineages [29,79-81]. In humans, 98% of V1R-like sequences identified in the genome are pseudogenes; in chimpanzee, there are no intact V1R-like sequences (100% pseudogenes) [29]. VNO-mediated pheromone perception declined in humans and Old World monkeys, coincident with the evolution of trichromatic color vision and dominance of the primate visual system [82]. But, even in rodents, the V1R pseudogene content, at least as compared to their OR pseudogene content, is high (~45% and ~50% in mouse and rat, respectively) [10,25,29]. Therefore, dynamic processes of gene death (excessive pseudogenization and gene deletion) as well as gene birth (by duplication and positive selection) have played significant roles in shaping rodent V1R repertoires.

### Cluster organization in the genome

**Figure 4:**
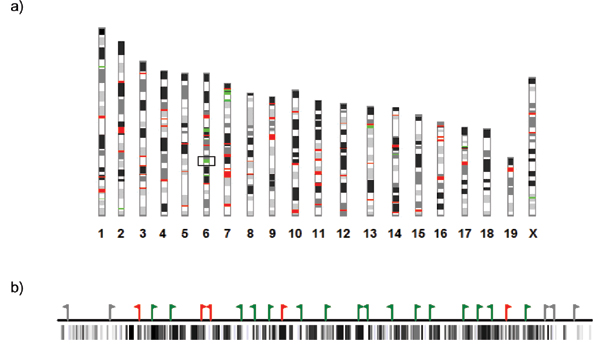
**Olfactory and vomeronasal receptor clusters in the mouse genome. a) **Chromosomal distribution of mouse OR and V1R genes. OR genes are on most chromosomes, with large clusters on chromosome 2, 7, 9, 11 containing the majority of OR genes. The major V1R gene clusters are located on chromosome 6, 7, 13 and 17. Figure adapted from [10], Copyright (2003), with permission from Elsevier. **b) **Schematic map of a V1R cluster located on mouse chromosome 6 (chromosomal location indicated by an open rectangle on ideograms shown in Fig. 4a) showing intact V1R genes (green flags), V1R pseudogenes (red flags), and flanking non-V1R genes (gray flags). LINE1 retrotransposon populations are indicated by vertical black lines below the map (as shown in the UCSC Genome Browser: ). The density of LINE1 populations is much higher within the V1R cluster than in surrounding flanking regions.

The large, diverse repertoire of mammalian odorant receptors is organized in clusters at various chromosomal locations in the genome (Fig. 4a). Generally, these clusters are arrays of closely related genes, reflecting an evolutionary history of recent tandem duplications. OR and V1R clusters are compact: OR coding sequences are single, ~1-kb coding exons [1], and they are densely packed with an average of ~25 kb spacing between neighboring coding regions [47,69]. In general, these clusters are rarely interrupted by non-OR/V1R genes (e.g., [19,52]) and occupy repeat-rich (especially Line1-rich) regions of the genome [19,26,52](Fig. 4b). We will return later to these attributes of OR and V1R clusters – compact, gene-poor and repeat-rich – when we consider the possibility that gene co-regulation is related to genome organization.

OR clusters are variable in size, and some ORs are unclustered. The largest human OR locus contains 116 ORs [13] and the largest mouse locus contains 244 ORs [76]. In humans, nearly half of the OR repertoire, including the entire set of Class I ORs, is present in two super-clusters on chromosome 11. Human OR genes are found at >100 loci and on all chromosomes except 20 and Y [19], and therefore are more dispersed than mouse OR genes, which are found at ~50 loci [44,47]. Much of this difference is due to the recently duplicated subtelomeric and pericentric clusters in humans [83]. It might be tempting to speculate that the subteleromeric/pericentric human OR clusters are simply neutral duplications of non-functional genes, however, the incidence of pseudogenes within these clusters is not higher than in other chromosomal locations [13].

Rodent VR genes also reside in well defined clusters in the genome [29]. Overall, ~94% of rodent V1Rs are located within clusters. In contrast, V1R repertoires in dog, chimpanzee, and human are much more dispersed: ~40% of V1Rs in these species are isolated in the genome [29]. Interestingly, analysis of intact versus pseudogene distributions suggests that isolated V1Rs in rodents and other mammals are more likely to be pseudogenes [29]. This observation hints of selective pressures to maintain functional genes together in the genome. We speculate that clustering might be favored in order to organize ORs and V1Rs within genome regulatory domains, a possibility we discuss in the following sections.

## Co-regulation of odorant receptor repertoires

In the previous sections, we have discussed the evolution of odorant receptor repertoires. Although there is evidence of fixation of some ancestral functions, such as Class I ORs in mammals [19,84] or specific OR orthologs between mouse and human (e.g., [47,85,86]), this evolutionary history can be described as "volcanic" – frequent gene duplication, gene deletion, recombination, gene conversion, pseudogenization, and positive selection have shaped very different repertoires between and within species. Rapid evolution is also a hallmark of multigene families of the immune system, like MHC gene families [87], which contrasts the evolutionary history of metabolic, regulatory, or developmental genes that tend to be fixed. The olfactory system, like the immune system, functions via interaction with the environment, and thus, must evolve at pace with changes in these environments. Also, since different species live in different niches (with different immune and olfactory requirements), there exists selective pressures to modulate both systems concurrent with speciation. In the olfactory system, the result is an ever-changing and widely distributed repertoire of odorant receptor genes in the genome.

Regulatory and developmental processes, as mentioned, are well buffered from the environment and generally, there is strong selection against "re-inventing" these systems in each species. For example, the assembly of a functional olfactory system is likely to be a conserved developmental process in mammals. Thus, we arrive at an interesting paradox. How might fixed regulatory processes used in the development of the olfactory system accommodate ever-changing odorant receptor components? Specifically, as we will next discuss, the assembly of a coherent olfactory system depends on the mutual co-regulation of odorant receptor repertoires in the genome. Thus, this paradox can be restated in a gene regulatory context: how does the presumably fixed odorant receptor regulatory process keep pace with the dynamic evolution of its target genes? Such a regulatory process would seem to be flexible, to accommodate new target genes, and involve genome-wide surveillance, to accommodate the variability in genome location of these target genes.

### The central organizing principle in the olfactory system is based on OSN specialization

The sense of smell begins in the sensory organ, the nose or VNO, where inhaled odorant molecules interact with odorant receptor proteins expressed on the surfaces of sensory neurons. Upon binding an odorant, a receptor signals the presence of the odorant through its associated G-protein, which eventually stimulates an action potential in the responding neuron [7,9]. How does the brain interpret what the organism is smelling, given the complexity of odorant mixtures and the sheer numbers of odorant receptors involved in the sensory process? The emerging view, based on several lines of study in primarily mouse and rat, is that olfactory coding is combinatorial: sensory neurons are specialized to recognize a narrow range of odorant chemistry, and particular "smells" are coded by discrete combinations of sensory neurons responding to the mixture of odorants in that "smell" [88].

This central organizing principle presents two developmental challenges. First, how do sensory neurons become dedicated to particular chemical attributes? And second, how does the brain know which combination of sensory neurons are responding to a particular smell? The answer to the first question, which is the main focus of the remainder of this review, seems to be that the large repertoire of odorant receptor genes in the genome is transcriptionally regulated such that each sensory neuron expresses one, or perhaps a small number, of odorant receptors. In this way, each sensory neuron is specialized to the odorant binding capabilities of the receptor(s) it expresses. The answer to the second question, which is beyond the scope of this review, is that all sensory neurons that express a particular receptor are guided to a common, stereotyped target glomerulus in the olfactory bulb [89-91]. In this way, the precise combination of responding sensory neurons is represented by a precise pattern of glomeruli activity, which is the internal representation of a particular smell that the animal can recognize, remember, and respond to.

### Odorant receptor genes are expressed in a mutual exclusive way

The specialization of sensory neurons in the olfactory system is accomplished by mutual exclusive expression of odorant receptor genes; that is, each sensory neuron expresses ~one odorant receptor, and is therefore functionally specialized. Initial evidence for singular OR expression in OSNs was from *in situ *hybridization studies conducted in rodent olfactory epithelium. Each OR tested had its expression confined to topological zones in the epithelium, and within a zone, the OR is expressed in a "punctate" pattern (i.e., in a seemingly random collection of neurons) [92-95]. The fraction of cells transcribing any particular OR is approximately one in a thousand (~0.1%), which is predicted by the one neuron-one receptor hypothesis if there are ~1000 ORs in the repertoire. A more compelling line of evidence for singular OR expression comes from single-cell reverse transcriptase polymerase chain reaction (RT-PCR) experiments using degenerate primers that are capable of amplifying ~70% of all OR templates [96]. In these studies, only one OR template is amplified from individual sensory neurons. However, the false negative rate is high – more than half of the cells tested do not produce any product – indicating that these experiments might underestimate the actual OR content per cell.

Additional evidence for singular OR expression comes from observations that pairs of a OR genes/alleles do not co-express in individual OSNs. Allelic exclusion was demonstrated using allele-specific PCR [97], and monogenic expression is supported by double labeling experiments, in which distinct genes never/very rarely are observed to co-express in individual cells [98,99].

Like ORs, TAARS and V1Rs seem to express in a mutually exclusive manner [2][100]. There is also transgenic and *in situ* hybridization evidence for monogenic and monoallelic expression of V2R genes [5,6,37], however, a recent study found that members of at least one V2R subfamily violate the one neuron-one receptor allele rule [101]. Since TAAR, VR and OR genes do not share homology, and are expressed in different sensory neurons, it is presumed that the similar monogenic/monoallelic regulatory strategy has been independently adopted in these systems. There is no evidence for a common molecular mechanism underlying these similar regulatory features, although this remains an interesting possibility.

### How is singular OR expression accomplished?

How is a vast and ever-changing repertoire of OR genes, dispersed over numerous chromosomes, co-regulated such that only one allele of one gene is expressed in an olfactory sensory neuron, while keeping the remaining alleles silenced? This remarkable problem could be solved by either a deterministic mechanism, in which each cell is determined by its spatial and temporal context to express a particular OR, or a stochastic mechanism, in which for example, each OR is competing for a limiting transcriptional complex.

The most deterministic solution is one in which each cell expresses a specific combination of transcription factors that is sufficient to activate only one OR. This model has been largely dismissed because mutual exclusive OR expression occurs even between two identical transgene copies [98], which should be able to bind exactly the same combination of transcription factors. Nevertheless, there is compelling evidence that OR gene regulation is at least partially deterministic. Initial studies suggested the existence of four discrete spatial zones in the rodent olfactory epithelium, with any particular OR being confined to one of these four zones [94,95]. More recent studies suggest less defined zonal boundaries inside the epithelium – there may exist numerous segregated but partially overlapping zones along the dorsal-ventral axis of the MOE [102-106]. Moreover, there seems to be spatial bias within zones; for example, the P2 OR exhibits a bias for expression in the posterior margins of its epithelial zone [107]. Other sets of ORs are expressed in non-canonical (e.g., medial) zones of the olfactory epithelium [108]. In addition, class I ORs exhibit a common promoter organization and expression pattern [109]. Thus, there seems to be spatial "rules" for OR expression, implying the presence of transcription factors that specify position in the MOE. Less well understood are temporal properties of OR expression, since most rodent studies have been conducted on adult animals. Studies in zebrafish, however, suggest that OR expression is partially dependent on developmental stage [110,111]. In total, these observations suggest the presence of transcription factors that, in a deterministic way, establish constraints on which OR genes are able to be expressed at a given time and place during development. The continuous, partially overlapping expression zones along the dorsal-ventral axis hint that a gradient of one or more signaling molecules might be a deterministic force [103].

Deterministic mechanisms predict constancy in the number of OSNs expressing a particular OR from animal to animal. Mouse-to-mouse variability in the numbers of positive cells for various ORs that have been tested seems to be in the 5–10% range [112], suggesting that the animal is not pre-programmed to generate a precise number of OSN types, as would be predicted by absolute deterministic models. Moreover, ablation of OSNs expressing a particular OR gene does not lead to immediate re-population of the ablated OSN type, suggesting that OR choice during OSN regeneration is not regulated in a deterministic way [113]. Most importantly, as mentioned previously, a deterministic mechanism for singular OR expression, in which OR choice is determined by spatial and/or temporal transcription factors, does not account for why identical *cis *sequences fail to respond identically to these cues [98]. Thus, there would seem to be a stochastic, or undetermined, aspect to OR regulation.

A stochastic element to OR expression was first postulated based on the observation that individual ORs seem to express in random sets of neurons within a zone, with no obvious patterning (e.g., [94]). Monoallelic expression of maternal or paternal OR alleles also appears to be random from cell to cell [97]. These observations point to a model in which subsets of ORs, presumably constrained by deterministic forces, compete stochastically for a stable active state.

**Figure 5:**
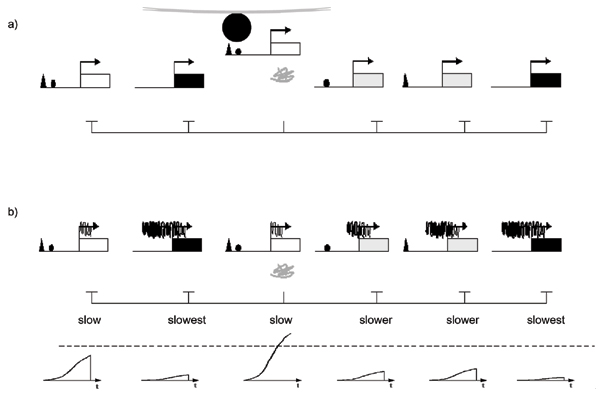
**Two stochastic models for mutually exclusive OR transcription.** Transcription factors (small triangles, circles) interact with *cis *regulatory sequences to generate OR gene loci that are competent for transcription (white versus gray/black rectangles). For example, OR genes might be more or less competent for transcription based on factors that specify geographic zone in the olfactory epithelium. **a) **Singular OR activating complex. A single transcriptional complex (large black circle) assembles at one physical location in the nucleus (e.g., nuclear structure or "H region"-like sequence in the genome [119, 120]), to which only one competent OR gene can stably associate. This model predicts that only one OR allele is physically able to be transcribed at once. In this model, feedback mechanisms might further stabilize/reinforce an OR-complex interaction or inhibit other OR loci from competing for the complex. **b) **Epigenetic regulation of OR genes. This model invokes a ground state of repression at OR loci. Deterministic factors specifying spatial or temporal information (small triangles, circles), or associated enhancers such as the "H region", reduce repressive chromatin states, thereby increasing rates of transcription. Because the overall transcriptional rates are nevertheless slow (even in the most activated loci), the likelihood of multiple ORs achieving a super-threshold level of protein expression (dashed line) is reduced. Kinetic profiles representing OR transcript levels over time (t) are shown at the bottom of the figure. Once a single OR achieves a super-threshold level of expression, a feedback inhibition mechanism silences other OR loci (represented by abrupt descent in kinetic profiles). Such a model requires that the selected OR locus be protected from this inhibitory process.

Two models for stochastic and mutually exclusive competition have been proposed. One model, based on the precedent of yeast mating type switching [114] and antigenic variation in *trypanosome *parasite [115], postulates that native OR loci compete for recombination/gene conversion into a single, transcriptionally competent locus (first proposed in [1]). Such a model now seems unlikely, based on two lines of evidence. First, RNA *in situ *experiments suggest OR transcripts are generated from native genomic locations [99]. Second, two groups have successfully cloned a mouse from differentiated olfactory sensory neurons [116,117], and in each case, the cloned mouse expresses the full repertoire ORs. Thus, OR gene choice is not irreversible, as might be predicted by changes in the actual DNA sequence. A second model, based on a precedent of mutually exclusive expression of the *trypanosome *parasite *variable surface glycoproteins *[118], postulates that OR loci compete for a single regulatory complex in the nucleus (first proposed in [85]; Fig. 5). The recent observation that active OR genes (but not inactive OR genes) interact in *trans *with a single locus, termed the "H-region", is compelling evidence for this model [119]. In this study, only one of the two "H region" alleles was observed to interact with the active OR locus; the second "H region" allele becomes differentiallly methylated, and this methylation is thought to render it inactive so that only one "H" is active per cell. In addition, the "H-region" had been previously identified as having strong OR enhancing activity when positioned near an OR promoter in transgene constructs [120]. This enhancement property, plus its apparently exclusive association with the transcribing OR locus in olfactory sensory neurons, makes the "H region" an excellent candidate locus for assembly of a single regulatory complex with which only one OR gene can associate per cell.

### Is mutual exclusivity absolute in OR transcription?

The above stochastic models predict that mutual exclusive expression is absolute – that cells cannot express more than one receptor at a time. In both flies and worms, mutual exclusivity is not absolute, and individual OSNs have been shown to co-express ORs [23,121]. But, as mentioned previously, the olfactory system in flies and worms bears no obvious homology to the mammalian olfactory system, and so there is no *a priori *reason to presume similar organizing principles. However, it has also been reported that some mouse V2R proteins co-express in individual vomeronasal sensory neurons [101]. Others report that some individual OSNs respond to chemically divergent odorants due to co-expression of divergent receptors (discussed in reference [122]). While most molecular and cell biological studies strongly suggest singular OR expression, isolated counterexamples that report OR co-expression (e.g., [98,122,123]) raise the possibility that the transcriptional mechanism underlying exclusivity is not necessarily absolute.

A recent discovery that production of a functional OR protein is required to ensure the "locking in" of receptor choice [120,124] lends further credence to these ideas. In these studies, it was shown that if the sensory neuron initially transcribes an OR gene that does not have an intact open reading frame, and thus cannot be translated into a functional OR protein, the sensory neuron will make another OR choice. Thus, it appears that post-translational feedback is an essential part of mutual exclusivity. Why might such a post-translational mechanism have evolved, if the transcriptional mechanism is itself designed to ensure mutual exclusivity? One plausible explanation is that feedback inhibition ensures that the sensory neuron will be productive, since the genome is populated with OR pseudogenes that might not have yet accumulated enough mutations to render their promoters non-functional [120,123,124]. It is also possible that such a post-translational feedback mechanism is needed for mutual exclusivity because mutual exclusivity is not ensured at the transcriptional level. Such an interpretation seems consistent with observations that an activated OR locus that cannot express a functional OR protein continues to be transcribed even after another OR locus is selected [120,124], suggesting that more than one OR promoter can indeed be active at once. Moreover, while an active OR gene is much more likely than an inactive OR gene to be associated with the "H region", a majority of expressing OR genes do not appear to be associated with the "H region" [119], suggesting that the OR-"H region" association might be transient and not absolutely required for ongoing OR transcription. In light of these observations, we propose an alternative stochastic model (described below) in which the "H region" functions to regulate epigenetic properties of an associated OR locus, as opposed to functioning as an ongoing activator of transcriptional machinery.

### An epigenetic model for mutually exclusive OR expression

As currently proposed, the "single activating complex" model predicts that only one OR locus can be transcribed at once, and that ongoing association with the complex is required to maintain OR expression throughout the life of the sensory neuron. As discussed in the previous section, this model does not appear to cleanly accommodate all experimental observations. If ongoing "H-region" association is mandatory, why do some cells expressing a particular OR not exhibit an association with the "H region" [119], and how is it possible that a non-functional OR locus whose coding region is deleted, presumably abandoned by "H" to select a second, functional OR locus, continues to be transcribed [124]? One possible explanation is that the "H region" is only transiently required. In this alternative model, the function of the "H region" might be to de-repress an OR locus and/or to protect the chosen locus from the "feedback inhibition" process; once de-repressed and the "feedback inhibition" process is completed, the requirement of "H" might be relaxed.

This model predicts that OR loci might vary in their dependence on the "H region" according to genome context. Interestingly, the mOR28 gene as well as other ORs in the mOR28 cluster, but unlike all other OR genes tested [107] require an "H region" positioned in *cis *in order to express from transgenes [120]; this requirement might reflect increased dependence on "H region" interactions (e.g., more frequent or prolonged association, as observed in [119], in order to achieve an adequately de-repressed state). It is also feasible that certain OR genes, by virtue of residing within more transcriptionaly permissive chromatin domains in the genome, might be able to express independently of an "H region" interaction. Alternatively, if the "H region" functions as part of the feedback inhibition step, as opposed to an initial choice step, then an association with the chosen OR locus would be required, but perhaps only transiently during the moment when the "feedback inhibition" signal is permanently silencing all other OR loci. With both interpretations, the proposed function of the "H region" is to regulate chromatin states – the former postulates that in order for an OR locus to be selected, chromatin de-repression by "H" is required; the latter postulates that in order to preserve activity during "feedback inhibition", the "H" is required to protect a locus from chromatin silencing. Finally, a third and completely different interpretation of the data cannot yet be ruled out: "H" might be one of numerous enhancers that can be utilized for OR selection, so that there is no ongoing and absolute requirement of any one of these enhancers (including "H"). Such an interpretation, however, requires an additional explanation for how only one of these enhancers becomes active per cell; one possibility is that one of multiple enhancers becomes active in the same way that only one of the two "H" alleles become active (via differential methylation).

Two of the above three interpretations of the "H region" data invoke a fundamental dependence on chromatin states as a "rate limiting" component of OR transcription. Whether as a means to reduce the probability of multiple ORs expressing prior to selection, or as a means to establish absolute silencing during "feedback inhibition", these models predict that OR genes, like imprinted genes or inactivated X-linked genes, can adopt a non-permissive/silenced chromatin state in cells that otherwise express these genes. We consider three circumstantial lines of evidence consistent with such an epigenetic model for OR regulation. First, as was described in previous sections on the evolution and genome organization of odorant receptor repertoires, OR and V1R genes occupy gene-poor and repeat-rich (especially Line1-rich) regions of the genome [19,26,52], as well as subtelomeric and pericentromeric regions of human chromosomes [46]. Given these features, it seems plausible that OR loci are in heterochromatic or more repressed regions of the genome that are more compatible with repeats than genes. Second, OR and V1R genes, like imprinted genes or X-linked genes, exhibit monoallelic transcription. As described above, allelic exclusion of imprinted genes and random inactivation of one X chromosome depends on differential DNA methylation patterns and the establishment of non-permissive chromatin on the silenced allele [125], and thus OR genes share regulatory attributes with other genes known to be regulated by epigenetic mechanisms. And third, a dominant position-effect has been observed to act on a transgene when integrated within an OR locus [108]. In this case, the transcriptional activity of an Olfactory Marker Protein (OMP) transgene is active only within a small region of the epithelium where the neighboring OR is expressed. This result suggests that the chromatin at this OR locus is generally non-permissive, and only permissive in the subset of cells where the OR is expressed.

With these observations and the "H region" data in mind, we propose an epigenetic model for OR regulation (Fig. 5B). In this model, chromatin at OR loci is generally repressive such that OR transcription is inefficient. In this way, the probability is reduced that multiple OR proteins achieve a super-threshold level of expression sufficient to trigger the "feedback inhibition" process. The probability for "multiple winners" might be further reduced if the "H region", or indeed other "H"-like enhancers, stochastically boost expression levels at select loci/locus. Some loci, for example, the mOR28 locus, might depend on such a boost in order to compete with less heterochromatic loci. Once an OR protein reaches a superthreshold level sufficient to trigger "feedback inhibition", the chromatin states at all other loci are fully silenced. The "H region" (or other "H"-like association), might then function to protect the locus from this silencing step. Such a model predicts that the "H region" might not be absolutely required for activation of all OR loci, that the probability of selection will depend on ground chromatin states, and that multiple ORs might routinely be transcribed per cell, albeit at very low (i.e., subthreshold) levels, early in this developmental process. The latter prediction seems consistent with the low-level, multi-OR transcription observed in non-OSN cell types [126]. Additional experiments will be required to clarify the role of the "H region", the molecular basis for stochastic properties of OR selection, and the importance, if any, of epigenetics in the monogenic and monoallelic expression of receptors in the olfactory and vomeronasal systems.

## Conclusion

The olfactory system meets niche- and species-specific demands by an accelerated evolution of its odorant receptor repertoires. As a result of a dynamic gene birth and death process, odorant receptor families vary significantly in size and quality between animals. In mammals, OR and V1R genes are co-regulated such that each sensory neuron expresses only ~one of the large repertoire of these genes. Singular receptor expression permits sensory neurons to be specialized to the odorant binding qualities of the single receptor expressed in each cell. This specialization of sensory neurons underlies olfactory coding, or the ability of the olfactory system to interpret and make sense of a complex odorant environment. The rapid evolution of odorant receptor genes, the sheer size of the repertoire, as well as their wide distribution in the genome, makes this co-regulatory task particularly daunting: what are the mechanisms of odorant receptor regulation that allow only one gene to be transcribed, while keeping the remaining large number of OR genes silenced? It appears that both deterministic and stochastic regulatory processes contribute to the expression patterns of odorant receptors. We have reviewed several models that have been proposed to account for mutually exclusive OR expression, as well as introduced a new hypothesis that invokes important epigenetic properties of the system. The discovery of a "trans activator", the "H region", is an eagerly awaited breakthrough. Future studies will elucidate how OR regulatory mechanisms accommodate a dynamic evolutionary history of gene birth and death, the importance of genome context and epigenetics, and how the "H region" and "feedback inhibition" collaborate in this fascinating problem of gene regulation.

## Competing interests

The authors declare that they have no competing interests.

## Authors' contributions

M.B.K. extensively researched the literature and compiled references, as well as making substantial contributions to the writing of this manuscript. R.P.L. contributed theoretical content, researched and compiled references, and organized and wrote the manuscript. Both authors read and approved the final manuscript.
